# Minimal clinically important differences for treatment of hallucinations in Parkinson’s disease and dementia with Lewy bodies

**DOI:** 10.1017/S0033291725000534

**Published:** 2025-03-24

**Authors:** Suzanne Reeves, Josef Mahdi, Matthew Appleby, Olga Zubko, Teresa Lee, Julie A. Barber, Kathy Y. Liu, John-Paul Taylor, Emily J. Henderson, Anette Schrag, Robert Howard, Rimona S. Weil, Hallucinations Working Group

**Affiliations:** 1 Division of Psychiatry, University College London, London, UK; 2 National Hospital for Neurology & Neurosurgery, London, UK; 3 Department of Statistical Science, University College London, London, UK.; 4 Campus for Ageing and Vitality, Newcastle University, Newcastle upon Tyne, UK; 5 Ageing and Movement Research Group, Bristol Medical School, University of Bristol, Bristol, UK; 6 Older People’s Unit, Royal United Hospitals NHS Foundation Trust, Bath, UK; 7 Movement Disorders Centre, Queen Square Institute of Neurology, University College London, Russell Square House, London, UK; 8 Dementia Research Centre, Queen Square Institute of Neurology, University College London, Russell Square House, London, UK

**Keywords:** Minimum clinical important difference, SAPS-H, UM-PDHQ, hallucinations, Parkinson’s disease, dementia with Lewy bodies, Delphi consensus

## Abstract

**Background:**

Hallucinations are common and distressing symptoms in Parkinson’s disease (PD). Treatment response in clinical trials is measured using validated questionnaires, including the Scale for Assessment of Positive Symptoms-Hallucinations (SAPS-H) and University of Miami PD Hallucinations Questionnaire (UM-PDHQ). The minimum clinically important difference (MCID) has not been determined for either scale. This study aimed to estimate a range of MCIDs for SAPS-H and UM-PDHQ using both consensus-based and statistical approaches.

**Methods:**

A Delphi survey was used to seek opinions of researchers, clinicians, and people with lived experience. We defined consensus as agreement ≥75%. Statistical approaches used blinded data from the first 100 PD participants in the Trial for Ondansetron as Parkinson’s Hallucinations Treatment (TOP HAT, NCT04167813). The distribution-based approach defined the MCID as 0.5 of the standard deviation of change in scores from baseline at 12 weeks. The anchor-based approach defined the MCID as the average change in scores corresponding to a 1-point improvement in clinical global impression-severity scale (CGI-S).

**Results:**

Fifty-one researchers and clinicians contributed to three rounds of the Delphi survey and reached consensus that the MCID was 2 points on both scales. Sixteen experts with lived experience reached the same consensus. Distribution-defined MCIDs were 2.6 points for SAPS-H and 1.3 points for UM-PDHQ, whereas anchor-based MCIDs were 2.1 and 1.3 points, respectively.

**Conclusions:**

We used triangulation from multiple methodologies to derive the range of MCID estimates for the two rating scales, which was between 2 and 2.7 points for SAPS-H and 1.3 and 2 points for UM-PDHQ.

## Background

Visual hallucinations are common in Parkinson’s disease (PD) and PD dementia (PDD), with a prevalence of approximately 40% (Aarsland et al., 2007), and are a core symptom of dementia with Lewy bodies (DLB) (McKeith et al., 2017). Although often initially benign, they can become distressing to patients, especially with loss of insight and disease progression (Fenelon, Mahieux, Huon, & Ziegler, 2000; O’Brien et al., 2020). Treatment is challenging as medications for motor symptom control can worsen or trigger hallucinations, and antipsychotics are linked with higher levels of morbidity and mortality (Weintraub et al., 2016). Cholinesterase inhibitors can improve symptoms, but do not wholly ameliorate hallucinations and thus further treatment is often required. Although pimavanserin has shown some efficacy (Cummings et al., 2014), this is not widely available outside of the United States. There is therefore a need for clinical trials of treatments for visual hallucinations in PD and related conditions, and these rely on questionnaires that quantify the frequency, severity, and impact of hallucinations to monitor treatment responses.

Clinical trials are conventionally powered, using sample size calculations, to detect clinically important effects as statistically significant. In order to do this appropriately, it is important to know the smallest clinically important effect for the chosen trial outcome. The minimum clinically important difference (MCID) is ‘the smallest difference in score in the domain of interest which patients perceive as beneficial and which would mandate, in the absence of troublesome side effects and excessive cost, a change in the patient’s management’ (Burback, Molnar, St John, & Man-Son-Hing, 1999; Jaeschke, Singer, & Guyatt, 1989).

One method of determining the MCID is an expert consensus approach, which involves seeking the opinion of an expert panel of clinicians who regularly treat the condition and/or experts with lived experience of the condition who are likely to have a greater understanding of what constitutes a clinically meaningful difference. As this value is subjective, opinions will vary and it is necessary to reach a consensus (McKenna, 1994). Typically, consensus-based methods are considered alongside statistical approaches to gain a balanced perspective (King, 2011; Revicki, Hays, Cella, & Sloan, 2008). Distribution-based methods rely on how widely the change in scores during a study varies between patients and determine the magnitude of change that would be required to be greater than what would be expected by chance. As a default, the MCID is conventionally set at approximately 0.5 of the standard deviation (SD) of change in outcome score between baseline and the primary endpoint (Norman, Sloan, & Wyrwich, 2003). The anchor-based approach is used to clarify the meaningfulness of a change in score by comparing it to an established, independent measure of clinically meaningful change (Revicki et al., 2008).

Ondansetron, a selective serotonin (5HT3) receptor antagonist licensed for use as an anti-emetic, is currently being evaluated in a multi-center study (Trial of Ondansetron as a Parkinson’s HAllucinations Treatment: TOP HAT trial) (ISRCTN51996779; NCT04167813). The primary effectiveness outcome measure is the change in Scale for Assessment of Positive Symptoms (SAPS) Hallucinations (H) (Andreasen, 1984) scores at 12 weeks, with the University of Miami Parkinson’s Disease Hallucinations Questionnaire (UM-PDHQ) (Papapetropoulos et al., 2008) as an important secondary outcome.

TOP HAT is the first trial to use the SAPS-H as the primary outcome and sample size calculations were based on previous trials of pimavanserin, which had included the SAPS-H as a secondary outcome (Cummings et al., 2014; Meltzer et al., 2010), and in which the standard deviation (SD) of baseline SAPS-H scores varied between 4.05 and 6.59 points. Based on an anticipated effect size of 0.5, a sample size of 172 participants (86 per arm) would allow TOP HAT to detect a treatment effect (difference in average SAPS-H between intervention and control) of 2 to 3 points, depending on the extent of variability of participant’s scores (assuming 90% power and a significance level of 5%).

## Aims

This study aimed to estimate MCIDs for SAPS-H and the quantitative items of the UM-PDHQ using consensus-, distribution-, and anchor-based approaches.

## Method

### Expert consensus

A Delphi survey was used to obtain expert opinions from clinicians with specialist knowledge in the management of PD, PD dementia (PDD), and/or DLB, including physicians (old age psychiatrists, neuropsychiatrists, neurologists, and geriatricians), nurses (Parkinson’s specialist nurses and research nurses) and other health professionals (occupational therapists, psychologists, and physiotherapists). The Delphi technique is an iterative multistep process, in which experts are asked to complete a series of anonymized surveys in order to reach a consensus (McKenna, 1994). The survey was developed using a free on-line tool (https://docs.google.com) and the responses were summarized and sent to the group at specific time points.

A panel of TOP HAT principal and sub-investigators were responsible for the study design, including the Chief Investigator (SR), investigators at University College London Hospital (UCLH) (RW, MA) and Luton and Dunstable Hospital (AS), and those with previous experience in conducting Delphi surveys (EH, RH, AS, and JPT). A clinician who was independent of TOP HAT investigators (JM) developed the survey, adapted case scenarios, and extracted the anonymized data.

Expert clinicians and researchers were approached by email via two routes:

1) Academic staff, who had been corresponding author on a peer-reviewed paper on the topic of Parkinson’s hallucinations, published in the last year. We identified relevant papers from a literature search (PubMed) of MESH terms ‘Parkinson disease’ and ‘Hallucinations’, limited by a 1-year date window.

2) All TOP HAT Principal Investigators and teams were invited to participate, and invitations to participate were also sent to relevant UK-wide professional networks (Association of British Neurologists Movement Disorders group, British Geriatric Society Movement Disorder Section; Royal College of Psychiatrists Old Age Faculty).

The email took for the form of a participant information sheet and explained what taking part would involve, and the fact that data would be anonymized.

### Delphi survey data collection

Each expert remained blinded to the identity of other participating experts and only those who completed the first round were invited to participate in subsequent rounds. JM had access to the email addresses of all who responded to the survey, but to no other identifiable information.

An introductory email provided information about the study and included a link to the online questionnaire, with a request to respond within 2 weeks of receipt of the invitation. Reminder emails were sent after 10 days. Expert clinicians and researchers who did not enter a particular round were not invited to participate in subsequent rounds.

Participants were asked to provide basic demographic information, their professional group (doctor, nurse, other), and setting (mental health, memory service, neurology, and medicine for older people) before accessing a link to a description of the rating scales and how they are scored. They were informed that the average baseline scores on SAPS-H in previous trials was 11 and this would equate to an average score of 8 on the quantitative items of the UM-PDHQ. Experts were then asked what they would consider to be the MCID for the SAPS-H and UM-PDHQ and given a choice of 1-, 2-, 3-, 4-, or 5- points. This was followed by eight clinical scenarios, each describing a person with PD or DLB and hallucinations, before and after drug treatment. The scenarios were anonymized descriptions of changes in frequency and/or severity of hallucinations following treatment, written by clinicians (RW, MA, AS) with experience of managing patients with hallucinations in the context of PD and/or DLB. They described differences ranging from 0 to 4 points in SAPS-H and 0 to 3 points in UM-PDHQ, but experts were not provided with information regarding scores. Experts were asked to give their opinion (yes, no, not sure) as to whether each scenario described a meaningful difference following drug treatment. We defined consensus as an agreement equal to or greater than 75% (Junger, Payne, Brine, Radbruch, & Brearley, 2017).

Experts who participated in round 1 were emailed a link to the second round and provided with the average scores of the group, and the proportion of respondents who chose a particular score. They were then asked to re-evaluate their estimation of both the MCID and their responses to clinical scenarios where a consensus had not been achieved, and were restricted to yes or no answers. Following a panel discussion (RW, MA, SR, RH, AS, EH, and JPT), the third round was restricted to case scenarios, and those seven panel members were excluded from taking part in this round.

### Feedback from experts with lived experience

Three focus groups (facilitated by SR and OZ), attended remotely using Zoom, were comprised of people with personal experience of hallucinations, either as a person with PD, PDD or DLB, or as a spouse, partner or family member, who had consented to participation. Each focus group followed an identical format: 1) introductions and sharing of experiences; 2) background to the research; 3) case scenarios presented to the group to obtain their opinions as to whether each scenario described a meaningful change, blind to the consensus opinions reached by expert clinicians and researchers in the Delphi survey; 4) the collective opinion of focus group members which was then compared to the consensus reached by expert clinicians and researchers to facilitate further discussion.

## Statistical analysis

### Delphi study

SPSS version 27.0 was used to analyze the data. Demographic data and responses to the survey were reported using number of responses (percentages). As MCID estimates were non-normally distributed, they were presented as median (25%–75% interquartile range, IQR).

### Distribution and anchor-based approaches

Statistical approaches used data from the first 100 PD participants in the TOP HAT clinical trial, blind to treatment status, which were downloaded as part of a planned interim analysis. The trial statistician calculated the estimates reported here and did not share any other additional data or summaries with the trial team. We obtained approval for sharing of these estimates from the trial Data Monitoring and Ethics Committee.

For the distribution-based approach, the SDs of change in SAPS-H and UM-PDHQ scores from baseline to 12 weeks (the primary endpoint) with 95% confidence intervals (CIs) were calculated across all subjects. For the anchor-based approach, the clinical global impression (CGI) - severity (S) scale (collected at baseline, week 2, week 4, week 6 and week 12) was used as an anchor. CGI-S is scored on a scale of 1–7 (1-normal; 2-borderline ill; 3-mildly ill; 4-moderately ill; 5-markedly ill; 6-severely ill; 7-among the most extremely ill) (Guy W, 1976), and a 1-point reduction, representing a one category improvement, is typically used as the MCID (Juniper, Guyatt, Willan, & Griffith, 1994).

As a moderate correlation (≥0.3) between an anchor and a clinical outcome measure is recommended for the appropriate estimation of an MCID (Revicki et al., 2008), we determined the Spearman’s rank correlation coefficient between the CGI-S and SAPS-H at each time point. We then estimated the MCID as the average change in SAPS-H corresponding to a 1-point change in CGI-S, using a 2-level linear mixed effects regression model to allow for repeated measurements with fixed effects for time and CGI-S. The same approach was used for UM-PDHQ.

## Results

### Clinician and researcher expert consensus

**Figure 1.**
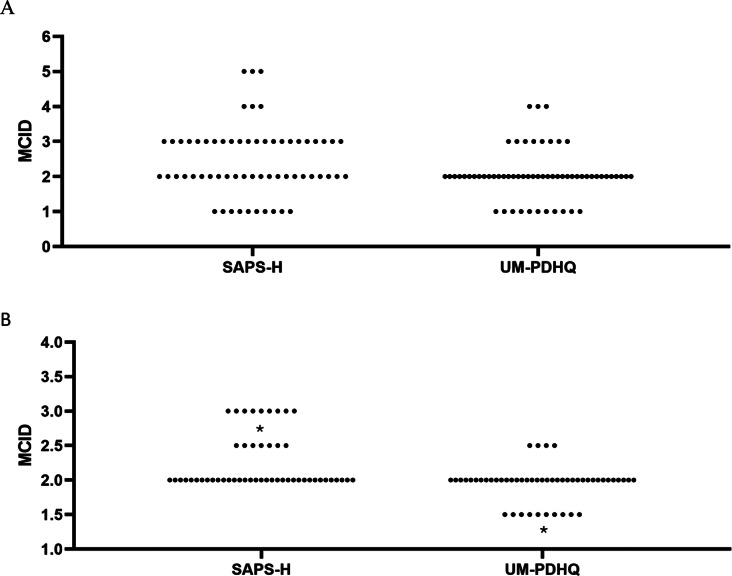
Delphi survey. *Note:* Scatterplots showing the distribution of opinions on the MCID for Scale for Assessment of Positive Symptoms Hallucinations (SAPS-H) and University of Miami Parkinson’s disease Hallucinations Questionnaire (UM-PDHQ) quantitative items in A) round 1 (n = 61) and B) round 2 (n = 51). MCID values determined by the distribution approach are shown as an asterisk.

There were 61 clinician and researcher participants in round 1, 51 in round 2, and 44 in round 3 as 7 subpanel members with prior knowledge of the case scenarios were excluded from taking part in the final round. Demographic characteristics, self-reported expertise, and specialty are shown in Table 1. In round 1, the median (IQR) MCID was 2 (1) points for SAPS-H. Opinions were largely split between 2 (37.7%) and 3 (36.1%) points, with a minority choosing 1-, 4-, or 5-points (16%, 4.9%, and 4.9%, respectively). The median (IQR) MCID was 2 (1) for UM-PDHQ, with the majority (63.9%) agreeing that the MCID was 2 points. Figure 1a shows the distribution of responses for SAPS-H and UM-PDHQ. A consensus was reached on 3 of the 8 vignettes (Table 2). There was consensus that a 0-point difference in SAPS-H and UM-PDHQ was not meaningful and that a 3- or 4-point difference in SAPS-H, corresponding to a 3- or 2-point difference in UM-PDHQ, respectively, was meaningful. Opinions were divided for case scenarios describing 1- and 2-point changes in SAPS-H and UM-PDHQ.

**Table 1.** Demographics of clinical and researcher expert Delphi survey participants

Category	Demographics	Round 1 (*n* = 61)	Round 2 (*n* = 51)	Round 3^a^ (*n* = 44)
		Number (%)	Number (%)	Number (%)
Sex	Male	34 (55.7)	27 (52.9)	25 (56.7)
	Female	27 (44.3)	24 (47.1)	19 (43.3)
Ethnicity	White (British or other)	46 (75.4)	39 (76.5)	32 (72.7)
	Asian/British Asian	9 (14.8)	7 (13.7)	8 (18.2)
	Other (African, Black British, Mixed, other)	6 (9.84)	5 (9.8)	4 (9.1)
Age, years	20–39	13 (21.3)	11 (21.6)	10 (22.7)
	40–59	41 (67.2)	35 (68.6)	31 (70.5)
	60+	7 (11.5)	5 (9.80)	3 (6.8)
Specialty	Elderly care	8 (13.1)	7 (13.7)	6 (13.6)
	Mental health	29 (47.5)	27 (52.9)	22 (50.0)
	Neurology	23 (37.7)	17 (33.3)	16 (36.4)
	Other	1 (1.64)	0	0
Profession	Doctor	52 (85.3)	44 (86.3)	37 (84.1)
	Nurse	4 (6.56)	2 (3.92)	3 (6.8)
	Other practitioner	5 (8.20)	5 (9.80)	4 (9.1)
Place of residence	England	53 (86.9)	45 (88.2)	36 (81.8)
	Scotland/Wales/NI	5 (8.20)	3 (5.88)	5 (11.4)
	Other	3 (4.92)	3 (5.88)	3 (6.8)

a 7 panelists with prior knowledge of Round 3 case scenarios were excluded from taking part. NI- Northern Ireland.

**Table 2.** Case scenarios and the responses from clinician and researcher experts for Rounds 1 and 2

Case scenarios				Number (%) who agreed the scenario described a meaningful difference	
Name^a^	Baseline score	Description of treatment effects	Number of points difference	Round 1 (*n* = 61)	Round 2 (*n* = 51)
Harry	SAPS-H 7 UM-PDHQ 8	↓ frequency of some visual hallucinations, but the emergence of others	SAPS-H 0 UM-PDHQ 0	No 51 (83.6) Yes 1 (1.6) Not sure 9 (14.8)	NA
Sheila:	SAPS-H 8 UM-PDHQ 11	↓ frequency of visual hallucinations, no change in distress or impact	SAPS-H 1 UM-PDHQ 1	No 21 (34.4) Yes 34 (55.7) Not sure 6 (9.8)	No 36 (70.6) Yes 15 (29.4)
Aaron:	SAPS-H 14 UM-PDHQ 13	↓ frequency, distress, and impact of visual and auditory hallucinations	SAPS-H 4 UM-PDHQ 3	No 3 (4.9) Yes 56 (91.8) Not sure 2 (3.3)	NA
Juan:	SAPS-H 10 UM-PDHQ 12	↓ distress and impact of visual hallucinations, no change in frequency.	SAPS-H 3 UM-PDHQ 2	No 2 (3.3) Yes 55 (90.2) Not sure 4 (6.6)	NA
^b^Evangeline:	SAPS-H 10 UM-PDHQ 13	Husband reports ↓ distress and impact, subjectively she reports no difference.	SAPS-H 2 UM-PDHQ 2	No 13 (21.3) Yes 36 (59.0) Not sure 12 (19.7)	No 3 (5.9) Yes 48 (94.0)
^b^Abdul:	SAPS-H 10 UM-PDHQ 11	↓ frequency of highly distressing visual hallucinations, no difference in distress or impact.	SAPS-H 1 UM-PDHQ 1	No 27 (44.3) Yes 27 (44.3) Not sure 7 (11.5)	No 41 (80.4) Yes 10 (19.6)
Jackson:	SAPS-H 15 UM-PDHQ 12	Wife reports ↓ impact of visual hallucinations.	SAPS-H 1 UM-PDHQ 1	No 22 (36%) Yes 25 (41%) Not sure 14 (23%)	No 35 (68.6) Yes 16 (31.4)
Mei:	SAPS-H 14 UM-PDHQ 12	↓ frequency and impact of hallucinations, but she still believes her family perceives her as ‘crazy’	SAPS-H 3 UM-PDHQ 2	No 24 (39.3) Yes 29 (47.4) Not sure 8 (13.1)	No 8 (15.7) Yes 43 (84.3)

a All names given are not the real names of patients.

b Scenario was subsequently presented to focus group participants with lived experience, who reached >75% consensus.

In round 2, the distribution of scores was summarized and the choice of MCID estimates restricted to 2-, 2.5- and 3-points for SAPS-H, and 1.5-, 2- and 2.5-points for UM-PDHQ. Although a consensus as defined by 75% agreement was not reached, a majority expressed the opinion that the MCID was 2-points for SAPS-H (68.6%) and for UM-PDHQ (72.5%). Figure 1b shows the distribution of responses for SAPS-H and UM-PDHQ. Consensus was achieved on three of the remaining five scenarios (Table 2): 94% agreed that a 2-point difference in SAPS-H and UM-PDHQ scores was meaningful, and 84.3% agreed that a 3-point difference in SAPS-H, which corresponded to a 2-point difference in UM-PDHQ was meaningful; the majority agreed that a 1-point difference in SAPS-H and UM-PDHQ was not meaningful in three case scenarios, two of which failed to achieve the 75% threshold for a consensus.

A panel discussion was held to discuss the approach to round 3, as experts had reached a consensus that the MCID was 2- points for both scales. A decision was made that round 3 would be comprised solely of case scenarios representing a 2-point difference in SAPS-H and UM-PDHQ to investigate the stability and consistency of their opinions. Case scenarios differed in relation to the initial frequency, distress and impact of symptoms, and whether the difference was based on a patient or caregiver account. A consensus was reached for all 4 cases (Table 3).

**Table 3.** Case scenarios: Round 3

Case scenarios describing a 2-point difference in scores on each scale			
Name^a^	Baseline score	Description of treatment effects	Number (%) who agreed the scenario described a meaningful difference (*n* = 44)^b^
Schlomit:	SAPS-H 11 UM-PDHQ 10	Subjective ↓ frequency and impact of distressing hallucinations	No 2 (4.5) Yes 42 (95.5)
^c^Samira:	SAPS-H 8 UM-PDHQ 7	Subjective ↓ frequency of hallucinations, ↓ in associated falls.	No 2 (4.5) Yes 42 (95.5)
Felix:	SAPS-H 10 UM-PDHQ 11	Subjective and informant based account, ↓ frequency and impact of hallucinations.	No 7 (15.9) Yes 37 (84.1)
Ruth:	SAPS-H 9 UM-PDHQ 11	Informant based account, ↓ frequency and impact of hallucinations	No 6 (13.6) Yes 38 (86.4)

a All names given are not the real names of patients.

b 7 panelists with prior knowledge of Round 3 case scenarios were excluded from taking part.

c Scenario was presented to focus group participants with lived experience, who reached >75% consensus.

### Experts with lived experience

Of the 16 people with lived experience who attended the focus groups, 8 (4 PD, 2 PDD, 2 DLB) had experience of hallucinations, 5 of whom attended with their spouse or family member (including the 4 with a diagnosis of dementia); and there were 2 former caregivers of husbands with DLB. There were 11 (69%) women and 11 (69%) were White British. When presented with two case scenarios describing a reduction in frequency and distress or impact of the hallucinations (reflecting a 2-point reduction in SAPS-H and UM-PDHQ), a consensus was reached that this was meaningful, regardless of whether the impact was reported by the person experiencing them or their caregiver. However, it was acknowledged that, where there is a mismatch between the person’s subjective experience and their spouse or caregiver, this should be explored further to establish the extent to which mood or memory deficits may be impacting. A consensus was reached that a case scenario describing a reduction in the frequency of distressing hallucinations (1-point reduction in each scale) was not meaningful as the impact (unpleasantness, emotional resonance) of hallucinations is more important than their frequency. When asked for final reflections, one attendee emphasized the importance of the person’s experience saying ‘*if a patient says it’s changing their lives, it is meaningful, even if it is a 1 point change’.*

### Distribution- and anchor-based approaches

Of the 100 participants with PD included in the interim analysis, the mean age ± standard deviation (SD) was 73.3 ± 7.1 years, and 64 (64%) were male and 88 (88%) were White British. Baseline scores were 12.6 ± 5.5- points and 11.5 ± 2.2- points for SAPS-H, UM-PDHQ (shown in Table 4). The SD of change between baseline and 12-week score SDs of change was 5.3 (95% CI 4.5–6.2) for SAPS-H and 2.6 (95% CI 2.2–3.1) for UM-PDHQ. Using the default of 0.5 SD of change, the MCID calculated using the distribution-based approach was 2.6-points for SAPS-H and 1.3-points for UM-PDHQ (shown in Figure 1b).

**Table 4.** Patient characteristics and distribution- and anchor-based results using data for first 100 TOP HAT trial PD participants

Baseline characteristics	
Age (years), mean (SD) (*N* = 100)	73.2 (7.1)
White British, number (%) (*N* = 100)	88 (88.0%)
Male, number (%) (*N* = 100)	64 (64.0%)
SAPS-H total score, mean (SD) (*N* = 98)	12.6 (5.5)
UM-PDHQ quantitative items, mean (SD) (*N* = 92)	11.5 (2.2)
CGI-S scale, mean (SD) (*N* = 100)	4.7 (0.8)
Distribution based approach: Change in scores (12 weeks -baseline)	
SAPS-H (*N* = 85)	
SD of change (95% CI)	5.3 (4.6 to 6.2)
0.5 SD of change	2.7 (2.3 to 3.1)
UM-PDHQ (*N* = 72)	
SD of change (95% CI)	2.6 (2.2 to 3.1)
0.5 SD of change	1.3 (1.1 to 1.6)
Anchor-based approach (based on mixed model using all repeated measurements):	
SAPS-H (*N* = 100)	
Estimated increase in SAPS-H per unit increase in CGI-S (95% CI)	2.1 (1.6 to 2.5)
UM-PDHQ (*N* = 97)	
Estimated increase in UM-PDHQ per unit increase in CGI-S (95% CI)	1.3 (1.0 to 1.6)

SD = standard deviation CI = confidence interval

Correlations between CGI-S and the two outcome measures were >0.3 at all time points, ranging from 0.49–0.60 for SAPS-H and 0.47–0.67 for UM-PDHQ, indicating that the anchor-based approach was appropriate. The mixed effects regression model estimated that a 1-point improvement in CGI-S would correspond to a 2.1-point (95% CI 1.6–2.5) reduction in SAPS-H and a 1.3-point (95% CI 1.0–1.6) reduction in UM-PDHQ.

## Discussion

This study aimed to obtain consensus on what should constitute the MCID for SAPS-H and UM-PDHQ. In the Delphi survey, we sought opinions from researchers and academic staff with expertise in PD and/or DLB, from a representative range of specialists (elderly care, mental health, neurology), who would typically be involved in the management of hallucinations. After two rounds, the majority agreed that the MCID was 2-points both for SAPS-H and UM-PDHQ, but a 75% consensus was not achieved; 31% of experts expressed the opinion that the MCID for SAPS-H would be greater than this (2.5- or 3-points) and 20% of experts felt that a 1.5-point change in UM-PDHQ would be meaningful. When subsequently presented with case scenarios representing a 2-point difference on either scale that corresponded to reduction in symptom frequency and impact, a very high level of agreement and a consensus (84% to 96% agreement) was reached that this would be meaningful. Experts with lived experience agreed (80% or greater agreement) with expert clinicians on case scenarios describing 1- and 2-point differences.

When a distribution approach was used to determine the MCIDs for SAPS-H (2.7- points) and UM-PDHQ (1.3- points), estimates were higher and lower, respectively, than the overall consensus reached by clinicians and experts with lived experience. They were, however, in line with those of a proportion of expert clinicians in the first two rounds of the survey as 31% felt that a 2.5- or 3-point change in SAPS-H would be meaningful and 20% expressed the opinion that a 1.5-point change in UM-PDHQ would be meaningful. Using the anchor-based approach, the estimated MCID for SAPS-H (2.1-points) was closer to the consensus approach and for UM-PDHQ (1.3-points) was closer to the distribution-based approach although 95% CIs overlapped.

It is important to reflect on reasons for the range of MCID estimates. When a distribution-based approach was used, there was greater variability in change scores on SAPS-H than UM-PDHQ and the estimated MCIDs were higher (SAPS-H 2.7) and lower (UM-PDHQ 1.3), respectively, than the consensus reached by clinicians and experts with lived experience. This may be partly explained by the properties of the two scales. SAPS-H measures the frequency and severity of hallucinations of several modalities (visual, auditory, olfactory, and tactile hallucinations) and assigns equal weight to each modality (maximum score 5 for each item), whereas the UM-PDHQ includes a single question on the types of hallucinations experienced (2-points scored if >1 modality), and the remaining questions pertain solely to visual hallucinations (severity, frequency, duration, real/not real, etc.). It is thus possible that variability in the change scores of different types of hallucinations may have contributed to our findings.

The inclusion of data from both treatment and placebo arms also needs to be considered as it is possible that non-treatment effects in the placebo group contributed to the degree of variability in change scores. If so, we would anticipate this would have a greater impact on SAPS-H than UM-PDHQ. It was not possible to compare SDs of change in treatment and placebo arms as data collection is not complete and blinding needs to be maintained. This could be investigated in future analyses following trial completion. A final consideration is that consensus-based MCID estimates were limited to being whole numbers due to the challenges (and questionable relevance) of conveying a change corresponding to less than 1-point on the scale using a vignette.

Strengths of the Delphi survey include the involvement of clinicians and researchers from a range of specialties (Coulter, Adams, & Shekelle, 1995) and an independent clinician, who developed and managed the online tool, to ensure anonymity was preserved. As a result, opinions were less likely to be influenced by knowledge of a person’s specialty, their level of seniority, or by the presence of more forceful personalities in the group (Drumm, Bradley, & Moriarty, 2022). The iterative approach of the Delphi survey enabled investigators to reflect and reconsider their responses through the process of controlled feedback without direct confrontation (Campbell & Cantrill, 2001). The vignettes, which described scenarios in which visual hallucinations were the predominant feature, were developed by clinicians directly involved in the clinical management of visual hallucinations and were involved in recruitment to the TOP HAT trial. The vignettes were therefore representative of the type and nature of visual experiences and of the ethnic and cultural diversity of patients who are seen in clinics, and focus group participants expressed their appreciation of this.

There are no guidelines on what constitutes achievement of consensus (levels of agreement ranging from 51% to 80% have been proposed) (Green, Jones, Hughes, & Williams, 1999; McKenna, 1994; Sumison, 1998) although the majority of health care studies have defined consensus as 75%–80% agreement (Junger et al., 2017). Although the defined threshold was not met when clinicians were asked to define the MCID in terms of number of points, the fact that consensus was consistently achieved for vignettes describing a difference of 2- or more points is a valuable indicator of the stability of responses when asked to assess clinically relevant scenarios (Crisp, Duffield, Adams, & Nagy, 1997). Involving those with lived experience of hallucinations in the content of PD and DLB was an essential component of the study as, in addition to obtaining feedback on the case scenarios, important common themes were revealed (Draak, de Greef, Faber, Merkies, & PeriNom, 2019), including the importance of balancing patient and informant accounts, and the impact of mood and cognitive deficits on the person’s subjective experience and their degree of insight.

There were some limitations to the Delphi process. These include low response rates to the initial email invitation and the low number of experts from outside the UK, which means that our findings may not be representative of the views of the international community. The preponderance of doctors perhaps reflects their closer involvement in the management of drug treatments for hallucinations compared to other specialties. The fact that most respondents were White British failed to accurately capture the ethnic and cultural diversity of clinicians and academics in this field.

The consensus-based approach we employed involved asking participants if a certain magnitude of change would be meaningful. However, what this word means to a given individual might have varied, and it has been proposed that there are several types of meaningful change (Liu et al., 2023; Weinfurt, 2019). For example, some individuals may view a reduction in frequency, however small, to be meaningful, while others may only judge a change to be meaningful if it reduced their distress or improved function, and this outweighed personal costs and inconveniences. Given the sensitivity of people with PD and DLB to medication side-effects, experts could be asked their views on the minimum important difference that would be worth the ‘cost’ (including expense and potential side-effects) of prescribing existing antipsychotic drug treatments.

It must be noted that the statistical approaches were based solely on data from the first 100 participants with PD as these data were made available as part of a pre-specified interim analysis. It was not possible to share data on participants with DLB, and this and a full sample of participants with PD from the trial will need to be further explored when data collection has been completed to ensure statistical findings are more precise and generalizable to PD and DLB patient groups.

## Conclusions

We combined the evaluations of clinician and researcher experts and experts with lived experience with statistical-based approaches, to estimate a range of MCID values for SAPS-H and UM-PDHQ. Combined, our findings suggest that a difference of at least 2-points on SAPS-H and 1.3-points on UM-PDHQ could represent the MCID. The purpose of triangulating estimations of the MCID was not aimed at establishing a single threshold value upon which to base decision-making regarding clinical meaningfulness. Understanding the range of MCID estimates derived from different methodologies will add context and meaning to our findings. This information can also be used to help power clinical trials for future trials of hallucinations in PD. When data collection is finished, we aim to use a similar approach to estimate MCIDs in DLB participants and in the full sample of PD participants. Future analyses will also include separation of SDs of changes in treatment and placebo arms, alongside further exploration of anchor-based approaches, such as use of the CGI-Improvement (I) scale.

## Supporting information

Reeves et al. supplementary material Reeves et al. supplementary material

## Data Availability

Data are not available for sharing as recruitment to the TOP HAT trial is ongoing.

## Abbreviations

5HT3: serotonin type 3
DLB: dementia with Lewy bodies
MCID: minimum clinically important difference
PD: Parkinson’s disease
PDD: Parkinson’s disease dementia
SAPS-H: Scale for Assessment of Positive Symptoms-Hallucinations
SD: standard deviation
UM-PDHQ: University of Miami Parkinson’s disease hallucinations questionnaire
TOP HAT: Trial for Ondansetron as a Parkinson’s Hallucinations Treatment

## Supplementary material

The supplementary material for this article can be found at http://doi.org/10.1017/S0033291725000534.
