# Supplementary material for: Minimal clinically important differences for treatment of hallucinations in Parkinson’s disease and dementia with Lewy bodies
Source: Psychol Med. 2025 Mar 24;55:e93. doi: 10.1017/S0033291725000534 (PMC12080632; doi:10.1017/S0033291725000534)
Supplement: Reeves et al. supplementary material [file S0033291725000534sup001.docx]

**Supplementary information**

**Members of the Hallucinations Working Group**

Alison Yarnall, [alison.yarnall@ncl.ac.uk](mailto:alison.yarnall@ncl.ac.uk)

ORCID 0000-0002-3126-9163

Campus for Ageing and Vitality, Newcastle University, Newcastle upon Tyne NE4 5PL

Funding: Newcastle Biomedical Research Centre NIHR203309

Financial disclosures: Has received funding from MJFF, EU IMI, NIHR, Parkinson’s UK, Lewy Body Society

[ashwani.jha1@gmail.com](mailto:ashwani.jha1@gmail.com)

Ashwani Jha, UCL Queen Square Institute of Neurology, Department of Brain Repair and Rehabilitation<https://orcid.org/0000-0003-2835-6837> COI: AJ is supported by the Wellcome Trust and the National Institute of Health Research University College London Hospitals Biomedical Research Centre. AJ has received expenses from Merz to attend a scientific course.

[a.sommerlad@ucl.ac.uk](mailto:a.sommerlad@ucl.ac.uk)

Division of Psychiatry, UCL

ORCID: 0000-0002-8895-7055

No COIs

Alex Bailey

Leeds and York Partnership NHS Foundation Trust

<https://orcid.org/0000-0002-2488-8791>

No COIs

[brady.mcfarlane@southernhealth.nhs.uk](mailto:brady.mcfarlane@southernhealth.nhs.uk)

Brady McFarlane, Southern Health NHS FT

ORCID: 0009-0007-9617-5882

No COI

[brian.parsons@berkshire.nhs.uk](mailto:brian.parsons@berkshire.nhs.uk)

[charles.marshall@nhs.net](mailto:charles.marshall@nhs.net)

Charles R Marshall, Centre for Preventive Neurology, Queen Mary University of London

ORCID 000-0002-8227-2354

No relevant conflicts to declare

[chineze.ivenso@wales.nhs.uk](mailto:chineze.ivenso@wales.nhs.uk)

Chineze Ivenso, Aneurin Bevan University Health Board

ORCID number: 0009-0003-3690-2698

<https://orcid.org/0009-0003-3690-2698>

Conflicts of interest relevant to the publication AND any other COI over the past year: Received ad hoc payments for advisory roles with Biogen, Eisai and Eli Lilly

[chris.southwell@southerntrust.hscni.net](mailto:chris.southwell@southerntrust.hscni.net)

No COI

[e.joyce@ucl.ac.uk](mailto:e.joyce@ucl.ac.uk)

Eileen M Joyce,

Emeritus Professor of Neuropsychiatry, UCL Dept of Clinical and Motor Neuroscience, Queen Square Institute of Neurology. ORCID 0000-0003-0496-2844

 No COI to declare

[eduardo.fernandez.13@ucl.ac.uk](mailto:eduardo.fernandez.13@ucl.ac.uk)

Eduardo de Pablo-Fernández, Darent Valley Hospital. Dartford and Gravesham NHS Trust; Queen Square Brain Bank. UCL Queen Square Institute of Neurology

Orcid number <https://orcid.org/0000-0003-2834-2515>

No conflicts of interest

[edward.jones@york.nhs.uk](mailto:edward.jones@york.nhs.uk)

Edward Jones, Department of Elderly Medicine, Scarborough Hospital, York and Scarborough Hospitals NHS Foundation Trust

No COI

[Fabrizia.dantonio@uniroma1.it](mailto:Fabrizia.dantonio@uniroma1.it)

Fabrizia D'Antonio, Department of Human Neurosciences, Sapienza university of Rome, Italy

ORCID number is 0000-0002-0898-5938

No COI

frankie.o'shea@nhs.net

Frankie O'Shea, The National Hospital for Neurology and Neurosurgery (NHNN)

no COIs

[gemsabhishek@gmail.com](mailto:gemsabhishek@gmail.com)

Abhishek Lenka, Parkinson's Disease Center and Movement Disorders Clinic, Department of Neurology, Baylor College of Medicine, Houston, Texas, USA.

ORCID number: 0000-0003-0725-636X

[georgecrowther@nhs.net](mailto:georgecrowther@nhs.net)

George Crowther, Leeds and York Partnership NHS Foundation Trust.

Orchid ID: 0000-0002-5102-4593

[gill.livingston@candi.nhs.uk](mailto:gill.livingston@candi.nhs.uk)

GL is supported by University College London Hospitals’ National Institute for Health

Research (NIHR) Biomedical Research Centre and North Thames

NIHR Applied Research Collaboration and as an NIHR Senior Investigator and has grants

from NIHR PGfAR, NIHR HTA,  Alzheimer’s Association, Norwegian Research Council and Wellcome with

no COI with current work

[Orcid 0000-0001-6741-5516](http://orcid.org/0000-0001-6741-5516)

Division of Psychiatry, University College London, UK; Camden and Islington NHS Foundation Trust, London, UK. Let me know if you need more.

[j.huntley@ucl.ac.uk](mailto:j.huntley@ucl.ac.uk)

Jonathan Huntley, Department of Clinical and Biomedical Sciences, University of Exeter

ORCID number: 0000-0001-6304-6231

No COI

[jason.raw@nca.nhs.uk](mailto:jason.raw@nca.nhs.uk)

Jason M Raw, Northern Care Alliance NHS Foundation Trust

No COI

[jay.amin@soton.ac.uk](mailto:jay.amin@soton.ac.uk)

Jay Amin, Clinical and Experimental Sciences, Faculty of Medicine, University of Southampton, UK

ORCID: 0000-0003-3792-0428

COI

[jd.neuropsi@gmail.com](mailto:jd.neuropsi@gmail.com)

JD Jurgens, NHS Highland

ORCID: 0009-0009-2451-9003

[Jennifer.Foley@nhs.net](mailto:Jennifer.Foley@nhs.net)

Jennifer A. Foley, Department of Neuropsychology, National Hospital for Neurology and Neurosurgery, Queen Square, London & UCL Queen Square Institute of Neurology, Queen Square, London.

ORCID: <https://orcid.org/0000-0001-5083-2823>

No CoI

[jonathan.rogers@ucl.ac.uk](mailto:jonathan.rogers@ucl.ac.uk)

Jonathan Rogers, Division of Psychiatry, UCL, London, UK

ORCID number: <https://orcid.org/0000-0002-4671-5410>

CoI: Jonathan Rogers reports research funding from the Wellcome Trust and NIHR; royalties from Taylor & Francis; payment for reviewing from Johns Hopkins University Press; and speaker fees from the Alberta Psychiatric Association, Informed Research & Training Ltd., North East London NHS Foundation Trust and Vanderbilt Medical Center. He is a Council member for the British Association for Psychopharmacology and conducts expert witness work.

[joseph.kane@qub.ac.uk](mailto:joseph.kane@qub.ac.uk)

Joseph PM Kane, Queen’s University Belfast

ORCID 0000-0002-8479-9977

COI for publication None

COI past year: Honoraria for Lewy Body Academy and Eisai

[kathryn.slevin@nca.nhs.uk](mailto:kathryn.slevin@nca.nhs.uk)

Kathryn Slevin, Salford Royal Hospital – Northern Care Alliance NHS Foundation Trust

No COI

[lucy.strens@uhcw.nhs.uk](mailto:lucy.strens@uhcw.nhs.uk)

Lucy Strens, Consultant Neurologist, Department of Neurology, University Hospitals Coventry & Warwickshire NHS Trust

ORCID number – 0009-0003-4963-5103

No COI

[matthew.smith@bristol.ac.uk](mailto:matthew.smith@bristol.ac.uk)

Matthew D. Smith, University of Bristol/Royal United Hospitals NHS Foundation Trust

ORCID: 0000-0002-4685-375X

COI: I am currently funded by a PDUK clinical excellence network grant

[monty.silverdale@manchester.ac.uk](mailto:monty.silverdale@manchester.ac.uk)

Monty Silverdale, Department of Neurology, Manchester Centre for Clinical Neurosciences, Manchester Academic Health Science Centre, University of Manchester.

ORCID ID: 0000-0002-3295-6897

No COI

[n.mukadam@ucl.ac.uk](mailto:n.mukadam@ucl.ac.uk)

Naaheed Mukadam, Division of Psychiatry, UCL

ORCID is 0000-0001-8635-9521

No COIs

[pmbarbosa@me.com](mailto:pmbarbosa@me.com)

Pedro Melo Barbosa, Clinical Research Fellow at the Reta Lila Weston Institute of Neurological Studies, Neurologista at University of Sao Paulo
ORCID: <https://orcid.org/0000-0001-7220-1563>
no COI
COI over the past year: Speaker fees for Abbvie.

[r.gould@ucl.ac.uk](mailto:r.gould@ucl.ac.uk)

Rebecca L. Gould, Division of Psychiatry, UCL

ORCID ID: 0000-0001-9283-1626

No COIs

[rdesilva@nhs.net](mailto:rdesilva@nhs.net)

Rajith de Silva, Barking, Havering & Redbridge University Hospitals NHS Trust

ORCID: <https://orcid.org/0000-0002-1257-8913>

richard.ellis22@nhs.net

[rob.skelly@nhs.net](mailto:rob.skelly@nhs.net)

Rob Skelly, Department of Medicine for the Elderly, University Hospitals of Derby and Burton

ORCID no: 0000-0001-8695-3299

COI: nil to declare

[rochelle.hernandez@uhd.nhs.uk](mailto:rochelle.hernandez@uhd.nhs.uk)

Rochelle Hernandez, University Hospitals Dorset

No conflicts of interest

[sam.abraham2@wales.nhs.uk](mailto:sam.abraham2@wales.nhs.uk)

Sam Abraham, Betsi Cadwaladr University Health Board

ORCID number: [https://orcid.org/0009-0000-2297-8630](https://eur01.safelinks.protection.outlook.com/?url=https%3A%2F%2Fscanmail.trustwave.com%2F%3Fc%3D261%26d%3D2cXY5sjz-BMBtx1rtQjjCBePdWnHuZNwr7w4HjLAUw%26u%3Dhttps%253a%252f%252forcid.org%252f0009-0000-2297-8630&data=05%7C02%7Co.zubko%40ucl.ac.uk%7Ca1be63533bc54bd7c31f08dc80988461%7C1faf88fea9984c5b93c9210a11d9a5c2%7C0%7C0%7C638526639751020149%7CUnknown%7CTWFpbGZsb3d8eyJWIjoiMC4wLjAwMDAiLCJQIjoiV2luMzIiLCJBTiI6Ik1haWwiLCJXVCI6Mn0%3D%7C0%7C%7C%7C&sdata=PX6pVfg%2B5GvrVaua2AeM9%2FPdgbF8erCx5mcpYb8iB3E%3D&reserved=0)

No COI

[sheeba.suresh@esneft.nhs.uk](mailto:sheeba.suresh@esneft.nhs.uk)

Sheeba Suresh, PD Advanced Clinical Practitioner, Co-PI for the study, Ipswich hospital,

ORCID number 0000-0002-9118-2094.

[turnergf@btinternet.com](mailto:turnergf@btinternet.com)

Thanks for this message, I have now retired from clinical and research practice and therefore am happy to be listed only as a non author contributor.

Gill Turner, Retired Consultant Geriatrician, Southern Health NHS  Foundation Trust

[c.ballard@exeter.ac.uk](mailto:c.ballard@exeter.ac.uk)

Clive Ballard, Professor of Age Related Diseases, University of Exeter

ORCID:  orcid.org/0000-0003-0022-5632

No direct COI for this paper

Other COI  Honoraria from Acadia, Roche, Exciva, Suven, TauRx, Orion, E Lilly, Abbvie, BMS, EI pharma and Johson and Johnson over the last 3 years.

[Jan.coebergh@nhs.net](mailto:Jan.coebergh@nhs.net)

Dr Jan Coebergh, Department of Neurology, Ashford St. Peter’s Hospitals NHS Foundation Trust, Chertsey, England

No COI
